# A two-step resin based approach to reveal survivin-selective fluorescent probes†

**DOI:** 10.1039/d0cb00122h

**Published:** 2020-11-27

**Authors:** Andrew J. Ambrose, Nhan T. Pham, Jared Sivinski, Larissa Guimarães, Niloufar Mollasalehi, Paula Jimenez, Maria A. Abad, A. Arockia Jeyaprakash, Steven Shave, Letícia V. Costa-Lotufo, James J. La Clair, Manfred Auer, Eli Chapman

**Affiliations:** Department of Pharmacology and Toxicology, College of Pharmacy, University of Arizona Tucson AZ 85721 USA chapman@pharmacy.arizona.edu; School of Biological Sciences and Edinburgh Medical School, Biomedical Sciences, University of Edinburgh The King's Buildings CH Waddington Building 3.07 Max Born Crescent Edinburgh EH9 3BF UK manfred.auer@ed.ac.uk; Departamento de Farmacologia, Universidade de São Paulo São Paulo SP 05508-900 Brazil; Instituto do Mar, Universidade Federal de São Paulo Santos SP 11.070-100 Brazil; Wellcome Centre for Cell Biology, University of Edinburgh Edinburgh EH9 3BF UK; Xenobe Research Institute P. O. Box 3052 San Diego CA 92163-1052 USA i@xenobe.org

## Abstract

The identification of modulators for proteins without assayable biochemical activity remains a challenge in chemical biology. The presented approach adapts a high-throughput fluorescence binding assay and functional chromatography, two protein-resin technologies, enabling the discovery and isolation of fluorescent natural product probes that target proteins independently of biochemical function. The resulting probes also suggest targetable pockets for lead discovery. Using human survivin as a model, we demonstrate this method with the discovery of members of the prodiginine family as fluorescent probes to the cancer target survivin.

## Abstract 2

A dual-resin system was developed that united confocal-fluorescent imaging for hit identification and microscale NMR for hit isolation and structure elucidation. Using this approach, we identified two natural products that bound to the inhibitor of apoptosis protein, survivin.

## Graphical abstract

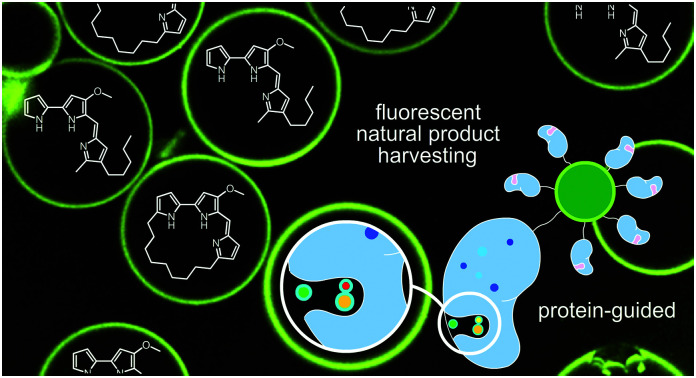

Nature contains a remarkable collection of spectroscopically active materials, including fluorescent compounds.^1^ Although many fluorescent dyes^2^ are derived from natural scaffolds,^3^ the translation of fluorescent natural products into biochemical probes remains particularly slow, with epicocconone providing a rare but excellent example.^4^ This disparity arises in part due to a disconnect that exists between the chemical and biological aspects of probe discovery. Here, we demonstrate how a two-resin system can function as a discovery platform for targeted proteins not amenable to high-throughput activity based screening. As shown in steps 1–4 of Scheme 1, this system applies a protein coated non-porous resin to screen and prioritize extracts. The prioritized extracts are then forwarded to a second, porous protein coated resin, which is used to isolate the active molecule from a mixture (steps 5–10, Scheme 1).

**Scheme 1:**
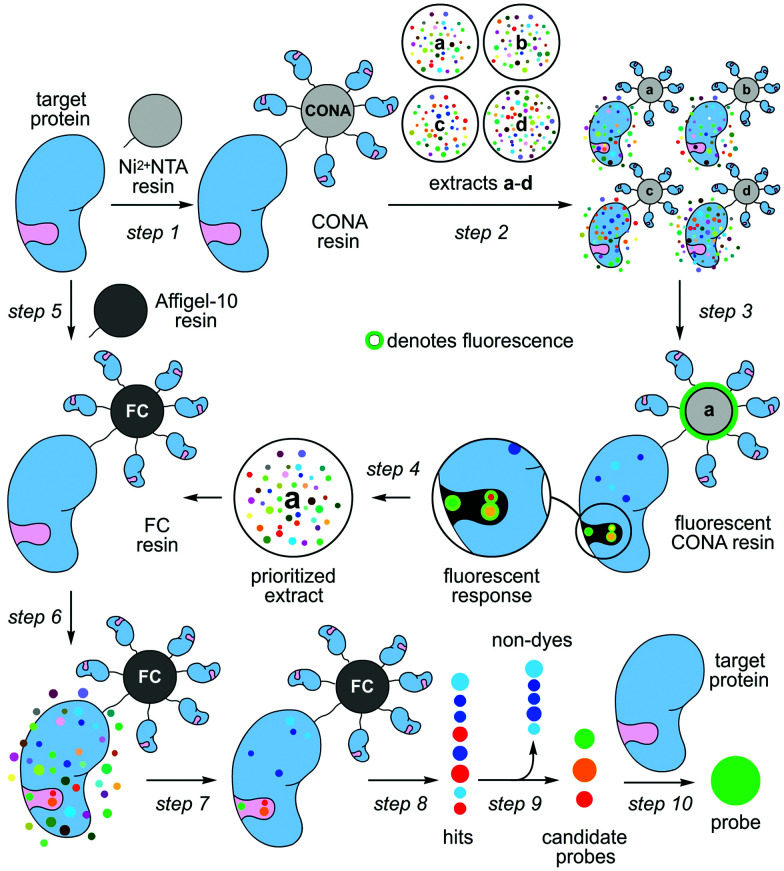
Target protein-guided hit prioritization and probe identification. Two different resins, a scanning confocal microscopy (CONA) resin and a functional chromatographic (FC) resin, are applied to screen extracts (a–d) for protein binders and isolate them. (step 1) The process begins by preparing Ni^2+^NTA resin loaded with a target protein (CONA resin). (step 2) The resulting CONA resin is presented with natural product extracts (a–d). (step 3) Compounds in target-protein active extracts bind to the target on the surface of the CONA resin. Upon excitation, fluorescent natural products on the CONA resin fluoresce as shown by a halo (green). (step 4) Extracts (a–d) are then scored based on the level of fluorescence observed on the CONA resin surface. (step 5) A second resin (FC resin) is prepared by covalently attaching the target protein to a porous resin (Affi-Gel 10 or 15). (step 6) Prioritized extracts from the CONA screen (steps 1–5) are then presented to the FC resin. (step 7) This resin is then washed with buffer to remove non-specific compounds. (step 8) The bound materials are eluted by denaturing the protein on the FC resin with an organic solvent (EtOH). (step 9) The resulting EtOH fractions containing hits are evaluated for fluorescence, and non-fluorescent hits are excluded (non-dyes). (step 9) The fluorescent hits are evaluated by capillary NMR, and if sufficiently pure are used as is. If not they are purified by HPLC or flash chromatography. (step 10) The binding of each candidate probe (hit) to its target protein is then validated.

A series of studies including those by our team (Chapman and La Clair)^5^ demonstrated that one can employ resin-bound protein as a vehicle to enrich for molecules that bind to a protein of interest (target protein). Using a complex mixture of molecules, this method represents a form of reversed-affinity or ‘functional’ chromatography (FC).^6^ In our studies, we demonstrated this process by identifying ligands to three different binding pockets on the ATPase p97; two of these molecules did not target p97's ATP-binding pocket, and could have been missed by conventional enzymatic screening.^5^

Parallel efforts led by the Auer laboratory developed microbead based screening technologies, initially for combinatorial chemistry^7^ and later for difficult enzymatic reactions^8^ and protein binding.^9^ Bead based scanning assays with confocal imaging readout, so called CONA assays, offer a series of advantages: they are miniaturized, versatile, extremely sensitive, multiplexed, quantitative, fast and can be automated.

Herein, we united efforts to explore a combination of CONA and FC to rapidly identify fluorescent natural product probes to an important oncology target with no known enzymatic function, the inhibitor of apoptosis (IAP), survivin. Fluorescent natural product probes offer two major advantages: (a) high-affinity, selective probes offer a means to study proteins in a cellular context by looking at subcellular localization while simultaneously studying the effects of a rapid inhibition of protein function; and (b) fluorescent probes, even with modest affinity and selective, provide a means to develop a fluorescent polarization assay that is amenable to high throughput competitive screening.

Survivin is the smallest member of the IAP family of proteins, and the first among the IAPs shown to have a function outside of caspase inhibition.^10^ In fact, survivin is likely incapable of directly inhibiting caspase activity, but instead binds a series of other proteins to carry out this anti-apoptotic function *in vivo*. In addition to these roles, survivin regulates signal transduction pathways associated with cancer.^11^ While suggested as a therapeutic target,^12^ drugging survivin or related IAPs has yet to reach fruition, and presents a drug discovery challenge we felt our strategy would be particularly suited to facilitate.^13^ Here, we develop an agnostic method to identify fluorescent ligands to survivin. The hope is that these tools will not only enable high-throughput screening efforts for IAP antagonists but also allow for untangling the complex physiologic actions associated with IAPs, including survivin.

Our studies began by screening a panel of 548 fluorescent marine microbial and sponge extracts to discover fluorescent survivin binders. We began by loading His_6_-tagged full length survivin to homogenous size Ni^2+^NTA agarose beads (step 1, Scheme 1; see Fig. S1, ESI† for survivin purification and purity analysis). The resulting resins were then presented with the fluorescent extracts (step 2, Scheme 1) and evaluated for fluorescence using CONA and image analysis (BREAD).^8,9^ Two hits were observed as given by the appearance of fluorescent halos. The brightest (strong halos in Fig. 1), an extract from *Actinomadura* sp. BRA 177 at 200× (25 μg mL^−1^) and 1000× (5 μg mL^−1^) dilutions from a DMSO stock generated a dose-dependent fluorescent response on the Ni^2+^NTA-resin with His_6_-survivin, but not on resins without survivin (steps 3–4, Scheme 1).

**Fig. 1:**
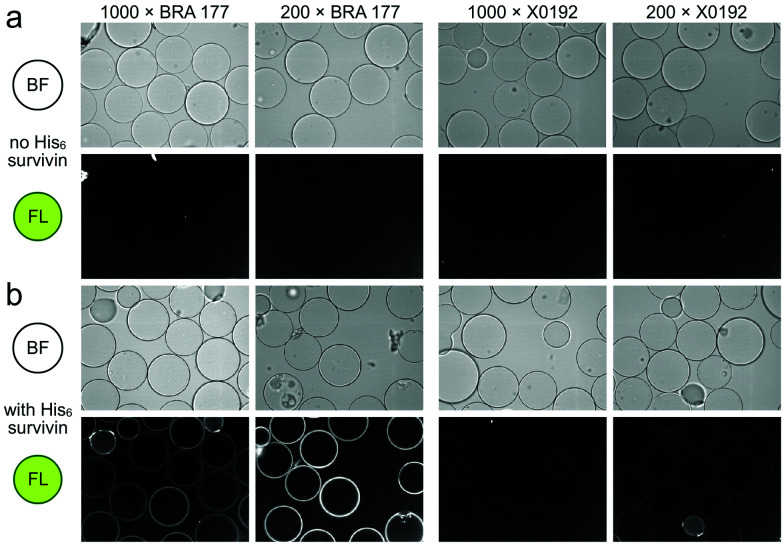
Confocal fluorescence scanning images arising from the incubation of unloaded Ni^2+^NTA resin or survivin-loaded CONA resin (Scheme 1) with 200 pmol (200 μL of 1 μM stock) of His_6_-survivin with extracts BRA 177 and X0192. (a) Brightfield (BF) and fluorescence (FL) images are shown for experiments using Ni^2+^NTA resin without survivin. (b) BF and FL images are shown for experiments with CONA resin. Two extract dilutions 1000× (high dilution) and 200× (low dilution) were compared to show fluorescence correlates with concentration. Fluorescence (FL) in both panels was collected by excitation at 561 nm and collecting emission at 585 nm. Fluorescence was not observed in control experiments using a non IAP protein, NusB/E (see Fig. S2, ESI†).

We then turned to the second or FC resin to isolate the active fluorescent material. Beginning with step 5 (Scheme 1), the FC resin was prepared by covalently attaching His_6_-survivin to Affi-Gel 10 resin. Using established methods,^5^ we were able to prepare a resin with 5 mg mL^−1^ (310 μM) of His_6_-survivin. A 300 μL aliquot of FC resin was then incubated with 1.6 mL of 10 mg mL^−1^ BRA 177 extract in pH 7.2 PBS containing 5% DMSO. After shaking at 4 °C (step 6, Scheme 1) for 12 h, the resin was washed with 1.6 mL of PBS (step 7, Scheme 1) and the bound materials were eluted by denaturing the protein in 200 μL of ethanol (EtOH) (step 8, Scheme 1). We then confirmed we captured the desired fluorescent material by screening the survivin-bound fractions from the FC resin with the CONA resin (steps 3–4, Scheme 1). As shown in Fig. 2b, we observed the same fluorescent response from the affinity-purified fraction as compared to controls (Fig. 2a).

**Fig. 2:**
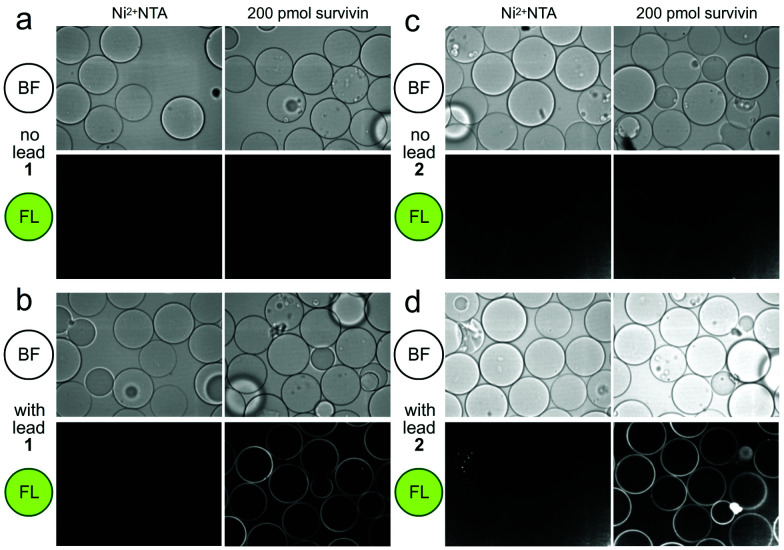
Confocal fluorescence scanning images of CONA resins presented with FC resin-bound fractions from the BRA 177 extract or X0192 fraction. (a) Control experiments using Ni^2+^NTA resin and the CONA resin containing 200 pmol (200 μL of 1 μM stock) His_6_-survivin. (b) Experiments using control Ni^2+^NTA resin and CONA resin containing 200 pmol survivin in the presence of 5 μM lead **1** from the FC resin bound extract of BRA 177 (see NMR of this fraction in Fig. 3a). (c) Control experiments using Ni^2+^NTA resin and CONA resin containing 200 pmol survivin. (d) Experiments using control Ni^2+^NTA resin and CONA resin containing 200 pmol survivin in the presence of 5 μM **2** from the FC resin bound extract of X0192 (see NMR of this fraction in Fig. 4b). BF indicates bright field images. FL in both panels was collected by excitation at 561 nm and collecting emission at 585 nm. Fluorescence was not observed in control experiments using a non IAP protein, NusB/E (see Fig. S2, ESI†).

Using a 1.7 mm probe for microscale NMR analysis, we were able to obtain a ^1^H-NMR spectrum from the affinity-isolated fraction evaluated in Fig. 2b. This spectrum contained aromatic protons between 5.5–7.5 ppm, typical of what one would anticipate from a fluorescent compound (Fig. 3a). We were then able to use NMR-guided flash chromatography to isolate 3.1 mg of pure cyclononylprodigiosin (**1**) from the BRA 177 extract. The assignment of **1** was further validated by high-resolution mass spectrometry (HRMS) returning *m*/*z* for C_23_H_30_N_3_O, [M + H]^+^ calculated: 364.2383; found: 364.2382. Full characterization of this extract was then conducted.^14^ While **1** was clearly evident in the FC resin-bound fraction (Fig. 3b), we also observed unsaturated fatty acid signatures (*, Fig. 3a) within this fraction, a common non-specific protein binder found during FC.^5^

**Fig. 3:**
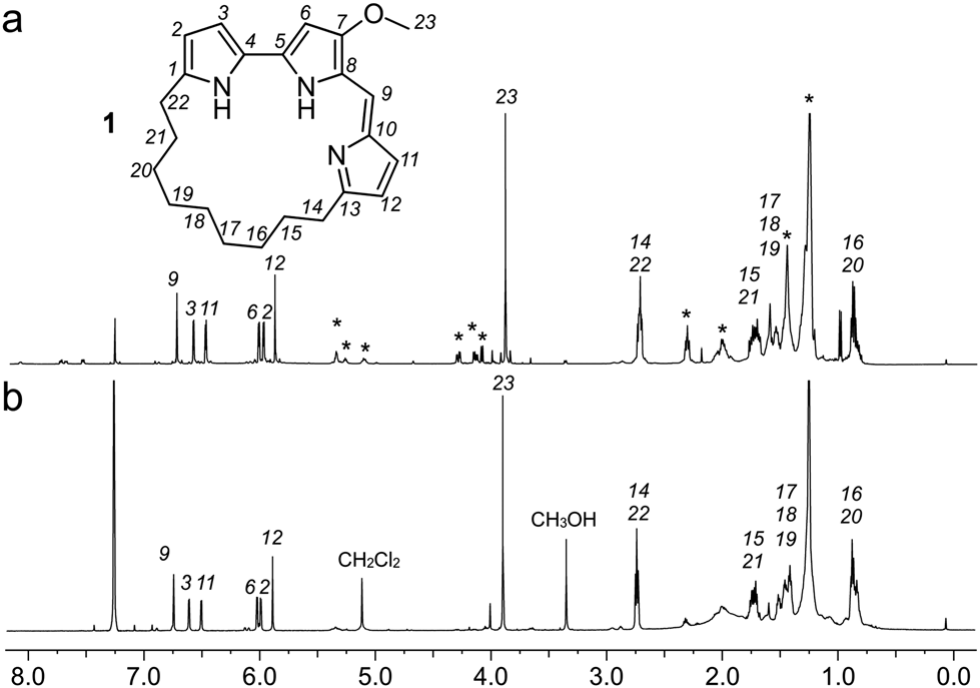
Identification of cyclononylprodigiosin (**1**) from the FC resin bound fraction of the BRA 177 extract. (a) A ^1^H NMR spectrum (600 MHz) collected from the survivin bound fraction in 35 μL of CDCl_3_. (b) A ^1^H NMR spectrum collected from pure **1** in CDCl_3_. Peaks from **1** are assigned by number. * denotes peaks that were not from **1**.

Overall, the NMR spectrum from **1** (Fig. 3b) displayed excellent correlation with that observed in the FC-enriched survivin-bound fraction (Fig. 3a) and gave a positive CONA result.^5^ Here, the union between CONA for hit screening and FC for compound enrichment and purification enabled rapid hit identification (CONA) and isolation (FC).

As a further confirmation of the power of this CONA–FC coupled approach (Scheme 1), we examined a second sample, X0192, a 1 : 1 EtOAc/hexanes fraction from an extract of the sponge *Xestospongia testudinaria*, that displayed weak fluorescence in the parent screen with the CONA resin (Fig. 1b) *versus* controls (Fig. 1a). Purification with the FC resin using the same methods as used for the BRA 177 extract, provided comparable fluorescence from the affinity-selected fraction (Fig. 2d) *versus* controls (Fig. 2b). Microscale NMR analysis again returned a ^1^H NMR spectrum that contained aromatic residues from the FC resin-bound fraction (Fig. 4b). Remarkably, this compound was only observed as a trace material in the X0192 fraction (Fig. 4a). Analysis of these data along with 1.2 mg of pure material purified by flash chromatography indicated that **2** was prodigiosin. This observation was further supported by HRMS data with an *m*/*z* for C_20_H_26_N_3_O, [M + H]^+^ calculated: 324.1998; found: 324.1987.

**Fig. 4:**
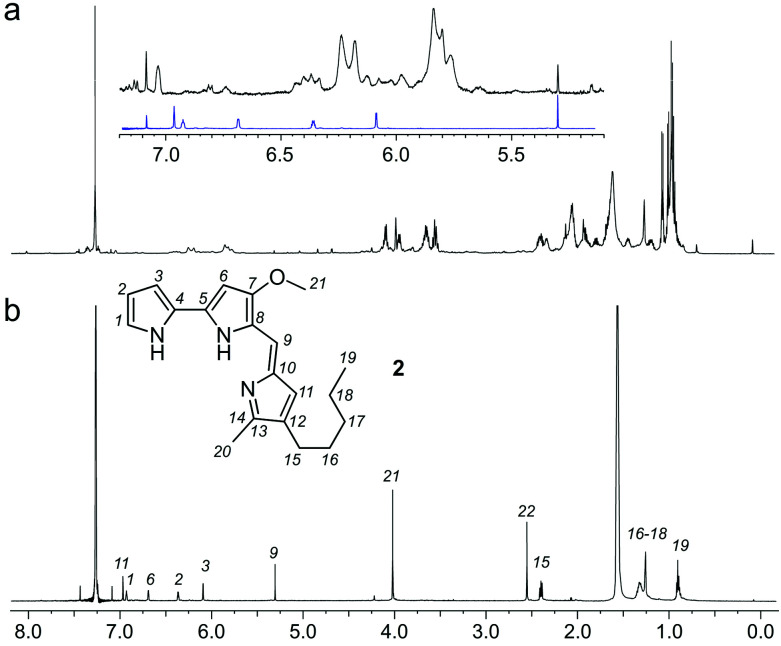
Identification of prodigiosin (**2**) from the FC resin bound fraction of the *Xestospongia testudinaria* X0192 fraction. (a) A ^1^H NMR spectrum (500 MHz) of the *X. testudinaria* fraction in CDCl_3_. (inset) Expanded region showing the NMR spectrum from **2** (blue) as compared to the X0192 fraction (black). (b) A ^1^H NMR spectrum (600 MHz) collected from the FC resin fraction containing **2** in 35 μL of CDCl_3_. Peaks from **2** are assigned numerically.

We then turned our attention to validate the binding of **1** and **2** to survivin in solution. We began by characterizing the fluorescence of **1** and **2** (Fig. 5a) in 20 mM HEPES, pH 7.5, 100 mM NaCl containing 2% DMSO. Absorption and emission wavelengths were determined to be *λ*_ex_ 534 nm and *λ*_em_ 556 nm for **1** and *λ*_ex_ 534 nm and *λ*_em_ 555 nm for **2**. However, we found that **2** exhibited a decay in fluorescence intensity once in solution (Fig. 5b and c). To ensure that this change in fluorescence did not interfere with the binding assay, the solution was left to equilibrate for 3–4 h until the fluorescence intensity stabilized. We next used Native-PAGE analysis to confirm the selective binding of survivin when compared to a similarly sized protein control, bromodomain 2 (BD2) of bromodomain containing protein 4 (BRD4). In addition, we saw survivin staining even when challenged with an *E. coli* lysate (Fig. S3, ESI†).

**Fig. 5:**
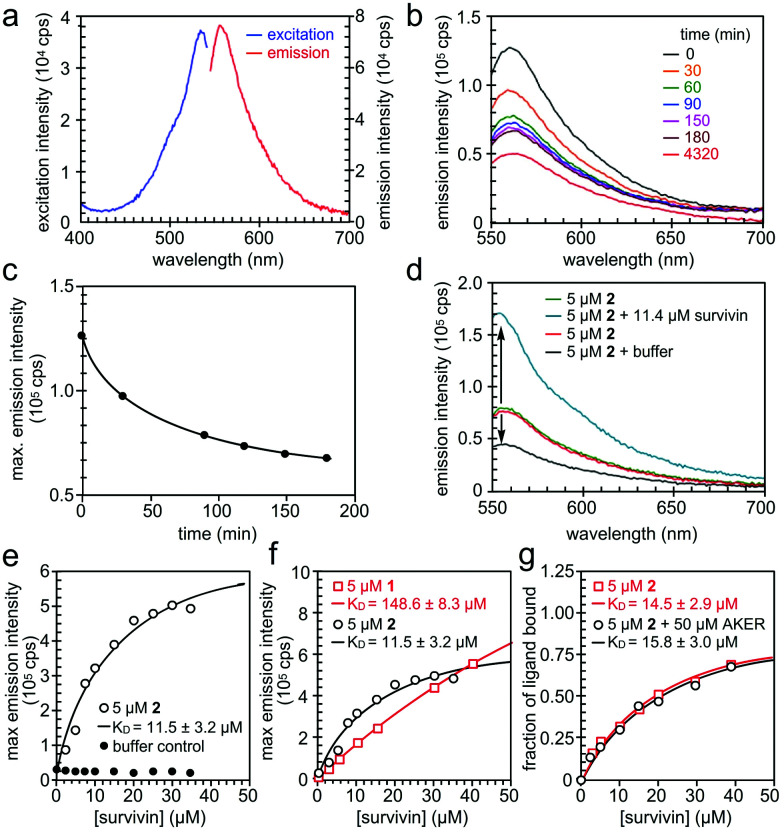
Spectral properties of prodigiosin **2**. (a) Fluorescence excitation and emission spectra of **2** in 20 mM HEPES, 100 mM NaCl, pH 7.5, 2% DMSO. (b and c) Reduction of fluorescence emission of **2** in the above buffer *versus* time passed from preparation of the solution. (d) The change in fluorescence emission intensity of **2** in the presence of His_6_-survivin. (e) The binding experiment of 5 μM of **2** and His_6_-survivin yielded a *K*_D_ value of 11.5 ± 3.2 μM. (f) The comparison of the dissociation constant *K*_D_ value of **1** and **2** with survivin. (g) The competition of **2** and a BIR domain ligand AKER (see ESI†).

When survivin was added to 5 μM of **2** in assay buffer, the fluorescence intensity increased significantly, without a spectral shift (Fig. 5d). However, when the same amount of assay buffer was added to **2**, a decrease in fluorescence was found due to dilution. This confirms that the increase in fluorescence intensity of **2** is due to binding to survivin, hence the change in the fluorescence properties of **2** can be used in a binding assay. The full binding curve for **2** and survivin, as well as for the buffer control, can be seen in Fig. 5e. A *K*_D_ value of 11.5 ± 3.1 μM was determined by fitting the fluorescence data with a 1 : 1 binding model, describing complex formation as a function of total survivin concentration. The same assay was repeated on **1**, with a *K*_D_ value of 148.6 ± 8.3 μM (Fig. 5f).

Finally, we performed a competition binding experiment of 5 μM of **2** and 50 μM AKER, a peptide known to bind at the BIR domain of survivin.^15^ The resulting *K*_D_ values in the presence and absence of 50 μM AKER were almost the same (Fig. 5g) indicating that **2** and AKER bind survivin independently, with no overlap of their binding sites. Such outcome produces a rather exciting proof of concept, while potentially revealing a new binding pocket for this IAP.

## Conclusions

This study demonstrates that a combination of a resin based CONA screen (Fig. 1 and 2) with FC enrichment/isolation (Fig. 3 and 4) returned two members of the prodiginine family of natural products from two distinct extracts as survivin binders. The spectral properties of prodigiosin (**2**) have a fascinating history as biological markers in the development of germ warfare and play a potential role in religious observations (bleeding statues and bread).^16^ Recent studies have shown that prodigiosin's fluorescence is environmentally sensitive, responding to DNA^17^ and protein^18^ binding. An elegant application of this method was recently demonstrated for the *in situ* quantification of prodigiosin biosynthesis.^19^

It is relevant to mention that survivin up regulation renders cells resistant to prodigiosin and, moreover, that prodigiosin and other prodiginine pigments have been shown to decrease expression of survivin (and other IAPs) in various human cancer cell lines.^20^ In fact, prodigiosin (**2**) was able to resensitize breast carcinoma cells to paclitaxel which, in turn, up regulates survivin making cells resistant to apoptosis.^21^ Our data strengthen the evidence on the central role undertaken by survivin in triggering the apoptotic process induced by **2**,^22^ potentially by directly targeting survivin.

Whilst prodigiosin (**2**) does not contain moieties which alert PAINS concerns, the presence of pyrroles of which there are three, does receive a special mention by Baell noting the hydrolysis of pyrrole containing compounds.^23^ Whilst often unstable due to polymerization to polypyrroles,^24^ the literature does allude to diverse biological activities for pyrrole containing compounds, such as antimicrobial, anti-viral, anti-malarial, anti-convulsant, anti-inflammatory, antipsychotic, anti-hypertensive, and finally anticancer agents.^25^ Additionally, a highly-substituted pyrrole moiety is also found in the approved drug atorvastatin (Lipitor). Using predicted oral-bioavailability as a measure of drug-likeness, the data suggests that **2** is a drug-like molecule for nearly all criteria;^26^ solubility: 56 μM, lipophilicity: log *P* 4.16, molecular weight: 323.43 g mol^−1^, polarity: 53.17 Å^2^ TPSA, flexibility: 7 rotatable bonds. The medical utility of this class is currently being explored through clinical trials on prodigiosin.^27^

Furthermore, the fact that prodiginines were found in two of the extracts, a microbial strain^14^ and a sponge known to contain prodiginines,^28^ suggests that mining the natural and synthetic diversity of this class could translate into probes for screening applications for survivin and related IAPs.^29^ Overall, this two-resin screening and enrichment approach, CONA–FC, provides a practical and cost-effective advance towards the identification of new fluorescent probes from diverse natural resources.

## Author contributions

N. T. P., M. A. A., A. A. J., M. A., and J. J. L. designed, conducted and evaluated the CONA assays, A. J. A., L. G., N. M., P. J., L. V. C.-L., J. J. L., and E. C., conducted the FC studies, P. J., L. V. C.-L., and J. J. L. isolated and elucidated cyclononylprodigiosin, J. J. L isolated and elucidated prodigiosin, N. T. P. and M. A. conducted the fluorescent assays, J. S. and E. C. ran the native PAGE analyses, S. S. evaluated the drug like properties, and all authors participated in the writing and editing of the manuscript.

## Conflicts of interest

There are no conflicts to declare.

## Supplementary Material

CB-002-D0CB00122H-s001
