# Supplementary material for: A two-step resin based approach to reveal survivin-selective fluorescent probes
Source: RSC Chem Biol. 2020 Nov 27;2(1):181–6. doi: 10.1039/d0cb00122h (PMC8342005; doi:10.1039/d0cb00122h)
Supplement: CB-002-D0CB00122H-s001 [file CB-002-D0CB00122H-s001.pdf]

Electronic Supplementary Material (ESI) for RSC Chemical Biology.
This journal is © The Royal Society of Chemistry 2020

Supporting Information

A Two-Step Resin Based Approach to Reveal Survivin-Selective Fluorescent Probes

Andrew J. Ambrose,[a] Nhan T. Pham,[b] Jared Sivinski,[a] Larissa Guimarães,[a,c,†] Niloufar Mollasalehi,[a] Paula Jimenez,[d] Maria A. Abad,[e] A. Arockia Jeyaprakash,[e] Steven Shave,[b] Letícia V. Costa-Lotufo,[c] James J. La Clair,[*,f] Manfred Auer,[*b] and Eli Chapman[*,a]

[a] Department of Pharmacology and Toxicology, College of Pharmacy, University of Arizona, Tucson, AZ, 85721, United States.

[b] School of Biological Sciences and Edinburgh Medical School: Biomedical Sciences, University of Edinburgh, The King's Buildings, CH Waddington Building 3.07, Mayfield Road, Edinburgh EH9 3BF, United Kingdom.

[c] Departamento de Farmacologia, Universidade de São Paulo, São Paulo, SP 05508-900, Brazil.

[d] Instituto do Mar, Universidade Federal de São Paulo, Santos, SP, 11.070-100, Brazil.

[e] Wellcome Centre for Cell Biology, University of Edinburgh, Edinburgh EH9 3BF, United Kingdom.

[f] Xenobe Research Institute, P. O. Box 3052, San Diego, CA 92163-1052, United States

[†] Current address: Universidade Federal do Piauí, Campus Senador Helvídio Nunes de Barros, Picos, PI, 64.607-670, Brazil

Correspondence should be directed to i@xenobe.org (J. J. L.), chapman@pharmacy.arizona.edu (E.C.), or manfred.auer@ed.ac.uk (M.A.)

Contents: Page

A. Recombinant protein preparation and purification. S2
B. Preparation of CONA resin and extract screening. S2
C. Confocal bead imaging (CONA). S2
D. Fluorescence intensity based binding assay S2–S3
E. Time-course fluorescence intensity measurements. S3
F. Preparation of functional chromatographic (FC) resin. S3
G. Functional chromatographic studies using the FC resin. S3
H. Flash chromatographic purification of **1**. S3
I. Flash chromatographic purification of **2**. S3
J. Capillary NMR analyses. S4
K. Data fitting. S4
L. Protein control. S4
M. Native PAGE analyses S4
Supporting Figure S1 S5
Supporting Figure S2 S6
Supporting Figure S3 S7
Additional Spectral Data S8-S12

A. Recombinant protein preparation and purification. Survivin full length was cloned into a pEC-His-3C vector as an N-terminally His_6-tagged protein. The survivin vector was transformed in *E. coli* BL21 Gold strain and grown in Super Broth media at 37 °C. Cultures were induced overnight at 18 °C with 0.35 mM IPTG. Cells were sonicated in lysis buffer (20 mM Tris•HCl pH 8, 150 mM NaCl, 25 mM imidazole and 2 mM 2-mercaptoethanol) and the His_6-survivin protein was purified by affinity chromatography using a 5 mL Ni^{2+}NTA column (GE Healthcare). The protein-bound column was washed with 10 column volumes of lysis buffer followed by 20 column volumes of high salt buffer (20 mM Tris•HCl pH 8, 1 M NaCl, 25 mM imidazole, 50 mM KCl, 10 mM $MgCl_2$, 2 mM ATP and 2 mM 2-mercaptoethanol). The protein was then eluted with 20 mM Tris•HCl pH 8, 150 mM NaCl, 400 mM imidazole and 2 mM 2-mercaptoethanol. Half of the pool of elutions was then cleaved with 500 µg of 3C protease overnight at 4 °C and the other half was left uncleaved. Subsequently, the concentrated His_6-survivin and survivin were further purified by size exclusion chromatography using a Superdex 200 Hiload 16/600 column or a Superdex 75 10/300 (GE Healthcare) pre-equilibrated with 20 mM HEPES pH 7.5, 100 mM NaCl and 5 mM DTT, respectively.

B. Preparation of CONA resin and extract screening. Ni^{2+}NTA agarose beads were prepared as described previously [Koszela, J.; Pham, N. T.; Evans, D.; Mann, S.; Perez-Pi, I.; Shave, S.; Ceccarelli, D. F. J.; Sicheri, F.; Tyers, M.; Auer, M. *BMC Biology* **2018**, *16*, 88]. Briefly, Ni^{2+}NTA agarose microbeads (Qiagen) were filtered using 100 µm and 120 µm filters (Corning or Millipore), sequentially, to obtain bead populations with bead diameters ranging between 100-120 µm. Sieved beads were washed thoroughly with binding buffer (0.3 M NaCl, 20 mM HEPES pH 7.5, 0.01% Triton X-100) and re-suspended to obtain 50% slurry. One microliter of filtered beads was used for each well of a 384-multi well plate (Swissci, PS384BG175). The volumes and amounts were scaled up according to the required number of wells.

The beads were incubated with 200 picomole (1 µM concentration in a final 200 µL volume/per well of beads) of His_6 tagged survivin in ice-cold binding buffer on a shaker at 1000 rpm for at least 20 min at 4 °C. After incubation, beads were extensively washed with reaction buffer (20 mM HEPES, 100 mM NaCl, pH 7.4, 5 mM DTT) and the volume of bead solution was adjusted to 10 µL per well. Beads loaded with survivin were distributed in 10 µL volumes into microplate wells and 10 µL of a pre-diluted extract solutions or pure prodiginine solutions were added and left to incubate on an orbital shaker at 200 rpm at rt for 1 h before imaging.

C. Confocal bead imaging (CONA). All imaging was performed on the Perkin Elmer OperaHCS system using a 20 × objective (LUCPLFLN, NA = 0.45). Detection was through one brightfield channel and one fluorescence channel with the following settings. Brightfield channel: excitation 690 nm LED at 70 % power, emission filter 690/70 and exposure time 160 ms. The 561 nm channel: 561 nm laser at 1500 µW, emission filter 585/35, exposure time 240 ms. All images were taken 25 µm above the well bottom. Other optional detection settings are: excitation 488 nm and emission 520/35 or 565/40 and excitation 640 nm and emission 690/70 nm. Autofluorescence from survivin was not observed using these wavelengths.

D. Fluorescence intensity based binding assay. All fluorescence experiments were carried out using the Fluorolog Tau-3 (FL-3-222) fluorescence spectrophotometer by Horiba Jobin Yvon with the following settings: detection photomultiplier HV at 950 V, excitation slit (in bandwidth) of 4 nm, emission slit at 5 nm, integration time at 1 second, Starna quartz 1 mL volume cuvettes (Type 29/9F). Excitation and emission spectra of prodigiosin **2** were obtained at 2 µM of **2** in 20 mM HEPES, 100 mM NaCl, pH 7.5, and 2% DMSO.

Survivin binding assay of prodiginines were performed in 5 µM of **1** or 5 µM of **2** in 20 mM HEPES, 100 mM NaCl, pH 7.5, 2% DMSO. The prodigiosin (**2**) solution was prepared and left for 3 h in the dark, transferred to two identical cuvettes and used in the titration experiment,

immediately. Volumes of survivin stock solution was added to one cuvette to obtain the required concentration while the same volume of buffer was added to the second cuvette as control. The content of the cuvettes was mixed and left to incubate for 5 min at rt before spectral measurements were taken. The total amount of volumes of survivin solution added to the cuvette was less than 5 % of the starting volume to minimize dilution.

E. Time-course fluorescence intensity measurements. The time course of fluorescence loss of **2** was determined by measuring the emission spectra of freshly prepared 5 μM of **2** in 20 mM HEPES, 100 mM NaCl, pH 7.5, 2% DMSO from 0 to 5.5 h and plotting the maximum fluorescence intensity *versus* time of incubation, the time difference between preparation of the solution and the measurement time point. Autofluorescence from survivin was not observed during these studies.

F. Preparation of functional chromatographic (FC) resin. A 15 mL conical tube was charged with 1 mL of Affigel-10 (BioRad) and the resin was washed with 3 × 10 mL of PBS pH 7.4 (50 mM NaPhos, 150 mM NaCl, pH 7.4). For all steps, the resin was collected by gentle centrifugation (1000 × g, 1 min). A 5 mL solution of His_6-survivin at 2.4 mg/mL in PBS pH 7.4 was cooled to 4 °C and then added to the resin suspended in PBS pH 7.4 (5 mL). The vial was mixed end over end for 3 h at 4 °C at which point Bradford analysis (ThermoFisherScientific) of the supernatant indicated that of the survivin protein was covalently-loaded on the resin as given by an OD_{595} <0.01. The resin was washed with 3 × 10 mL of PBS pH 7.4 at 4 °C and stored under PBS pH 7.4 (5 mL) and was used within 48 h of preparation.

G. Functional chromatographic studies with the FC resin. A 1.6 mL solution extract (10 mg/mL) in PBS pH 7.2 (1 mL) containing 5% DMSO was added to 300 μL of resin bearing 5 mg/mL of His_6-survivin in a 2 mL Eppendorf tube and shaken on an inversion rotator at 4 °C. After 12 h, the resin was by gentle centrifugation (1000 × g, 1 min). The supernatant was discarded and the resin washed three times with 1.6 μL of PBS (50 mM NaPhos, 150 mM NaCl, pH 7.4). After the third wash, bound molecules were eluted with 200 μL of EtOH. The EtOH fraction was transferred to a flame dried ½ glass vial, dried by airflow, and analyzed by capillary NMR analyses.

H. Flash chromatographic purification of 1. A sample of the BRB-117 (92.5 mg) was subjected to fractionation using 10 cm x 2.5 cm ID silica gel column (SiliaFlash P60, Silicycle) by eluting with 100 mL fractions: A (hexanes), B (2:1 hexanes/EtOAc), C (1:1 hexanes/EtOAc), D (1:2 hexanes/EtOAc), E (EtOAc), F (10:1 EtOAc/MeOH), G (5:1 EtOAc/MeOH), H (2:1 EtOAc/MeOH). The B fraction was collected and subjected to a second column to return 3.1 mg of cyclononylprodigiosin (**1**). Further detail on this isolation effort can be found in [Silva, A. E. T.; Guimarães, L. A.; Ferreira, E. G.; Torres, M. da C. M.; Silva, A. B. da; Branco, P. C.; Oliveira, F. A. S.; Silva, G. G. Z.; Wilke, D. V.; Silveira, E. R.; Pessoa, O. D. L.; Jimenez, P. C.; Costa-Lotufo, L. V. *J. Braz. Chem. Soc.*, **2017**, *28*, 465-474]. Copies of NMR spectra are provided at the end of this document.

H. Flash chromatographic purification of 2. A sample of the X0192 (42.9 mg) was subjected to fractionation using 10 cm x 2.5 cm ID silica gel column (SiliaFlash P60, Silicycle) by eluting hexanes to 1:1 hexane/EtOAc to return 1.2 mg of prodigiosin (**2**). This material was compared with a commercial sample (Cayman Chemical). Copies of NMR spectra are provided at the end of this document.

I. Capillary NMR analyses. NMR data were acquired with a Bruker Avance III 600 equipped with a 1.7mm cryoprobe. Chemical shifts were referenced using the corresponding solvent signals (δ_H 7.26 and δ_C 77.00 for $CDCl_3$, δ_H 3.31 and δ_C 49.0 for CD_3OD). The NMR spectra were processed using Mnova 11.0 (Mestrelab Research) or TopSpin 3.0 (Bruker Biospin) software.

J. Competition experiments. Competition experiments were performed with 5 μM of **2** in absence and presence of 50 μM of AKER, a known binder to the BIR domain of survivin with a K_D value of 6.2 μM [Jeyaprakash, A. A; Basquin, C.; Jayachandran, U.; Conti, E. *Structure* **2011**, *19*, 1625], was performed to investigate whether **2** shows an overlapping binding site with AKER. However, results shown in Fig. 5f suggest that **2** and AKER bind independently to survivin as the K_D for **2** remains unchanged in the presence of 50 μM AKER.

K. Data fitting. To determine the K_D the maximum fluorescence intensities of prodigiosins were plotted *versus* the total concentration of survivin. The data was fitted in GraFit (Erithacus Software) [Leatherbarrow RJ. GraFit Version 7. Horley, U.K: Erithacus Software Ltd.; 2009] using a 1:1 binding model exemplified by the solution of the quadratic equation describing signal increase linearly proportionally to complex formation as function of total survivin concentration with K_D and maximum fluorescence intensity as fitting parameters.

L. Protein control. To check for unspecific binding of **2** we immobilized His_6-NusB:NusE (NusB/E) (received from S. Knauer, University of Bayreuth, Germany) onto Ni^{2+}NTA and performed the binding experiment with 5 μM of **2**. No fluorescence ring beads were observed indicating that **2** does not bind to NusB/E complex, part of the anti-termination complex in bacteria [Drögemüller, J.; Strauβ, M.; Schweimer, K.; Jurk, M.; Rösch, P.; Knauer, S. H. *Sci Rep* **2015**, *5*, 1-14].

M. Native PAGE staining. To determine the specificity of **2** for survivin (16.5 kDa) over other proteins, we ran survivin and/or *E. coli* lysate, and BRD4-2 (bromodomain-containing protein 4, domain 2, MW 16.5 kDa) in 1× native sample buffer (3×: 3 mL glycerol, 6.4 mL H_2O, bromophenol blue, 0.6 mL 50× running buffer) in non-denaturing conditions over 12 h at 80V in 1× native running buffer (50×: 7.5g tris base, 36 g glycine, H_2O to 250 mL) on a 12% native gel (37.5:1 acylamide/bis-tris gel containing ammonium persulfate (APS), Tetramethyl-ethylenediamine (TEMED), Tris-base with pH 6.8 stacking and pH 8.8 resolving gels). After running, the native gel was stained in 10 μM **2** in water for 1 h, washed in water for 5 min twice, and visualized by fluorescence at λ_{ex} = 524 nm and λ_{em} = 572 nm) using gel scanner (Azure Biosciences 600).

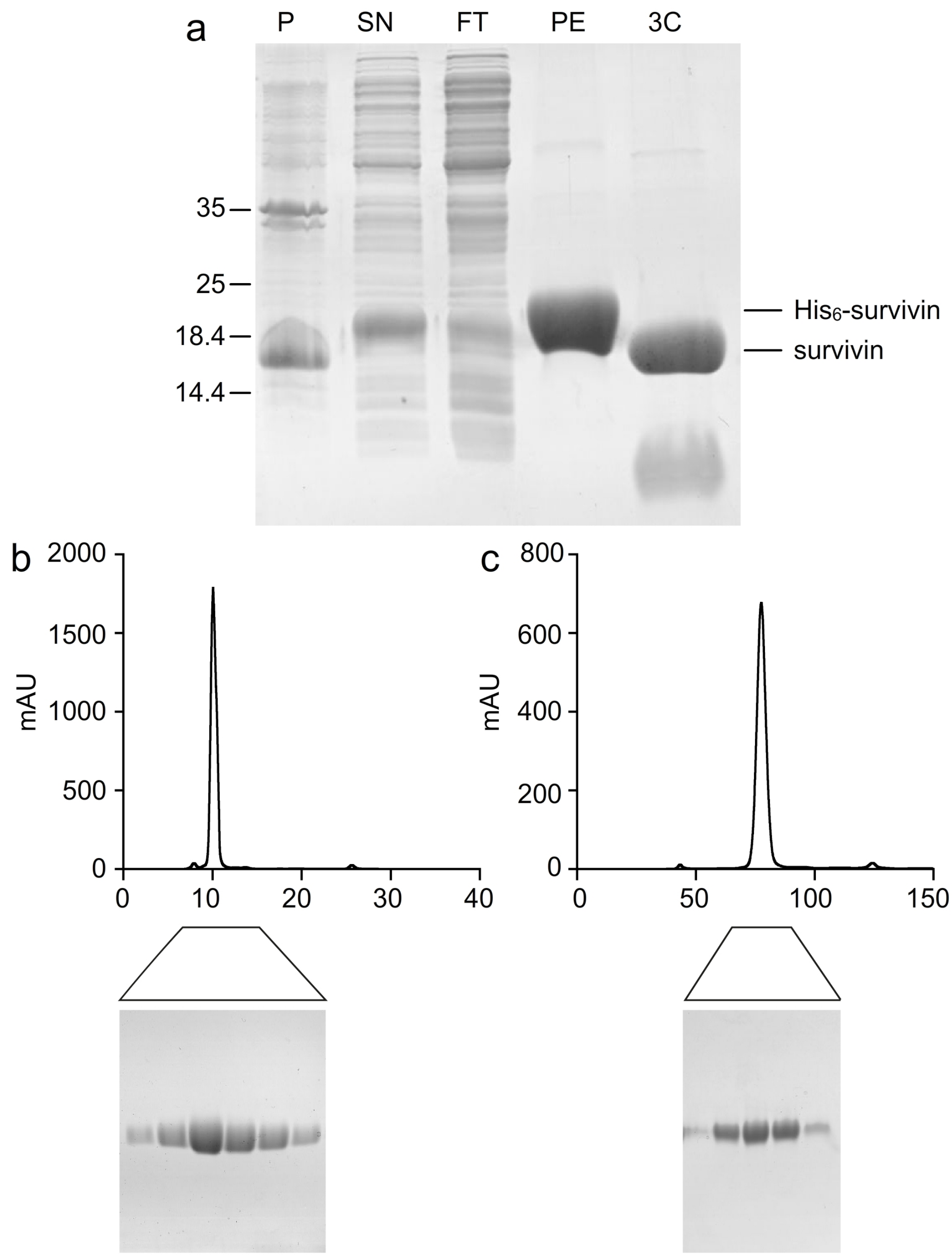

Supporting Figure 1*.* Protein purification. **a)** 15% SDS-PAGE analysis of Ni-NTA affinity purification steps for the His$_6$-survivin with P = pellet or insoluble fraction, SN = supernatant or soluble fraction, FT = flow through, PE = pool of elutions, 3C = 3C-cleaved protein. **b)** 15% SDS-PAGE analysis of size exclusion chromatography peak fractions of the His$_6$-survivin purification. **c)** 15% SDS-PAGE analysis of size exclusion chromatography peak fractions of the survivin purification.

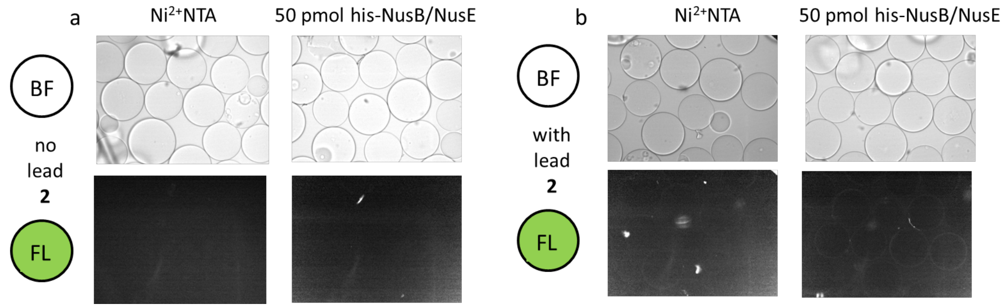

Supporting Figure 2. Selectivity evaluation using His_6-NusB:NusE (NusB/E) as unspecific protein control. **a)** blank Ni^{2+}NTA beads (left) and NusB/E loaded beads (right) in absence of lead compound **2**. **b)** blank Ni^{2+}NTA beads (left) and NusB/E loaded beads (right) in presence of 5 μM lead compound **2**.

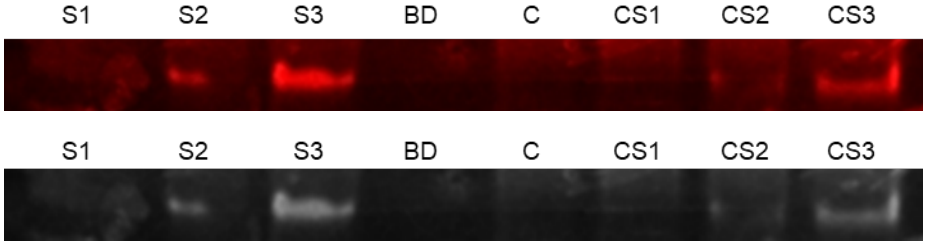

Supporting Figure 3*.* Native PAGE. 12% Native PAGE analysis of interaction of **2** for 16.5 kDa proteins survivin (S) and BRD4-BD2 (BD) as well as *E. coli* lysate (C) demonstrates specificity for survivin. Survivin alone (S1 = 25 µg, S2 = 50 µg, S3 = 75 µg), BRD4-BD2 alone (BD = 75 µg), *E. coli* lysate alone (C = 75 µg), or *E. coli* lysate plus increasing concentrations of survivin (CS1 = 75 µg *E. coli* lysate and 25 µg survivin, CS2 = 75 µg *E. coli* lysate and 50 µg survivin, CS3= 75 µg *E. coli* lysate and 75 µg survivin) were ran on a native gel, stained with 10 µM **2**, and visualized by fluorescence at λ_{ex} = 524 nm and λ_{em} = 572 nm.
